# Normothermic Machine Perfusion vs. Static Cold Storage for DBD Liver Transplantation in High‐MELD Recipients: A Single‐Center Retrospective Study

**DOI:** 10.1002/jhbp.70129

**Published:** 2026-05-08

**Authors:** Yuzuru Sambommatsu, Daisuke Imai, Kush Savsani, Mallika Datta, Junpei Tarashi, Jacob Hallesy, Samuel Wolfe, Isao Yokota, Seung Duk Lee, David A. Bruno

**Affiliations:** ^1^ Department of Surgery, Division of Transplant Surgery, Hume‐Lee Transplant Center Virginia Commonwealth University School of Medicine Richmond Virginia USA; ^2^ Department of Biostatistics Hokkaido University Graduate School of Medicine Sapporo Japan

**Keywords:** graft survival, liver transplantation, normothermic machine perfusion, organ preservation, postoperative complications

## Abstract

**Background and Purpose:**

While normothermic machine perfusion (NMP) has benefits over static cold storage (SCS), its efficacy in high‐acuity recipients with high Model for End‐Stage Liver Disease (MELD) scores, particularly using a back‐to‐base application model, is not well established. We aimed to compare short‐term outcomes between NMP and SCS in this cohort.

**Methods:**

This retrospective cohort study analyzed 215 adult recipients with a laboratory MELD score ≥ 25 who underwent isolated liver transplantation from donation after brain death (DBD) donors between July 2022 and June 2024. Patients were divided into SCS (*n* = 137; median MELD, 35) and NMP (*n* = 78; median MELD, 36) groups. Outcomes were compared using multivariable regression analyses.

**Results:**

NMP was associated with higher daytime surgery rates (88.5% vs. 23.4%) and lower peak alanine aminotransferase levels (median 298 vs. 653 IU/L). However, no significant differences were found in blood loss, early allograft dysfunction, acute kidney injury, comprehensive complication index, hospital stay, or 90‐day graft survival.

**Conclusions:**

In high‐MELD recipients of DBD grafts, a predominantly back‐to‐base NMP approach did not improve major short‐term clinical outcomes compared to SCS. Benefits of NMP appear to be highly context‐dependent across three axes—graft type, application model (upfront vs. back‐to‐base), and recipient acuity.

**Abbreviations**
AKI: acute kidney injury
ALD: alcohol‐related liver disease
ALT: alanine aminotransferase
AST: aspartate aminotransferase
BMI: body mass index
CCI: Comprehensive Complication Index
CI: confidence interval
CIT: cold ischemia time
DBD: donation after brain death
DCD: donation after circulatory death
EAD: early allograft dysfunction
EBL: estimated blood loss
FFP: fresh frozen plasma
HBV: hepatitis B virus
HCC: hepatocellular carcinoma
HCV: hepatitis C virus
ICU: intensive care unit
IQR: interquartile range
KDIGO: Kidney Disease Improving Global Outcomes
LDRI: Liver Donor Risk Index
MASH: metabolic dysfunction–associated steatotic hepatitis
MELD: Model for End‐Stage Liver Disease
NMP: normothermic machine perfusion
OPTN: Organ Procurement and Transplantation Network
OR: odds ratio
OR: operating room
PBC: primary biliary cholangitis
POD: postoperative day
PRS: post‐reperfusion syndrome
PSC: primary sclerosing cholangitis
RBC: red blood cells
SCS: static cold storage
SMD: standardized mean difference
Tbil: total bilirubin
UNOS: United Network for Organ Sharing

## 1 Introduction

Normothermic machine perfusion (NMP) of the liver has been rapidly adopted in the United States since its U.S. Food and Drug Administration approval in 2021. Numerous studies have reported improved post‐transplant outcomes compared with static cold storage (SCS). Specifically, previous studies have demonstrated that NMP reduces post‐reperfusion syndrome (PRS) [1, 2, 3, 4], reduces intraoperative blood transfusions [3, 5, 6] and lowers the incidence of early allograft dysfunction (EAD) [1, 2, 6, 7]. Furthermore, NMP has been shown to reduce the incidence of postoperative acute kidney injury (AKI) [6, 8], shorten both intensive care unit (ICU) and total hospital stays [3, 5, 6], and decrease ischemic biliary complications [2, 7].

However, these benefits of NMP have been primarily demonstrated within a specific clinical context. Early experiences were often part of clinical trials with strict inclusion criteria, which typically excluded the most severely ill recipients [1, 2, 4] and even in the real‐world setting, many centers have applied NMP selectively to grafts from extended criteria donors (ECD)–such as those from DCD, older, or steatotic donors, which are often allocated to recipients with low‐to‐moderate Model for End‐Stage Liver Disease (MELD) scores. Furthermore, many of these studies utilized portable, upfront NMP systems (e.g., TransMedics OCS), which are initiated at the donor hospital to minimize cold ischemia time (CIT) [2, 3, 5, 6, 7, 9].

In contrast, our institution's practice involves a different clinical scenario. We are a high‐volume center for severely ill recipients with high MELD scores, who typically receive grafts from standard criteria, donation after brain death (DBD) donors. Since July 2023, our center has adopted a back‐to‐base NMP model as its primary preservation method, where the liver is first transported to our center under SCS before being connected to the OrganOx Metra perfusion system. This approach results in a longer CIT compared with the upfront NMP model, but its impact on clinical outcomes remains unclear.

This discrepancy raises the question of whether the reported advantages of NMP can be extrapolated to our distinct patient cohort. Therefore, the objective of this study was to determine whether, in a cohort of high‐MELD recipients receiving DBD grafts, preservation with NMP demonstrates superior short‐term outcomes compared with SCS.

## 2 Patients and Methods

### 2.1 Institutional Normothermic Machine Perfusion Protocol

Our center first adopted the TransMedics OCS in 2019 on a selective basis for participation in clinical trials or to address logistical challenges. In July 2023, we shifted our preservation strategy by expanding NMP to near‐universal use and establishing it as our primary approach using the Organox Metra system in a back‐to‐base model, with the dual aims of improving operational feasibility (facilitating daytime transplantation and reducing overnight staffing demands) and potentially improving early graft and recipient outcomes. Comprehensive details regarding the establishment of our NMP program, including the specific indications for NMP and viability criteria, have been previously published [10]. Briefly, since July 2023, OrganOx Metra back‐to‐base NMP has been the default preservation at our center. When a local recovery surgeon was not available, we used the TransMedics OCS as an alternative. Conventional SCS was considered only as an exception when a standard‐criteria graft was allocated to a clinically straightforward recipient and the graft was expected to arrive in the morning, in which case transplantation proceeded under SCS without NMP (Table S1).

### 2.2 Study Design

This retrospective cohort study analyzed deceased donor liver transplants performed at Virginia Commonwealth University Hume‐Lee Transplant Center (Virginia, U.S.A.) between July 2022 and June 2024. This study was conducted in full compliance with the Declaration of Helsinki and the Declaration of Istanbul and was approved by the Institutional Review Board of Virginia Commonwealth University (HM20029955). Because of the retrospective design, the requirement for written informed consent was waived. This report followed the Strengthening the Reporting of Observational Studies in Epidemiology (STROBE) guidelines for observational studies [11]. This study consisted of three distinct analyses.

#### 2.2.1 Part 1: Institutional Trends

To evaluate institutional trends in response to a shift in our preservation strategy, we analyzed all 334 adult, deceased donor liver transplants performed during the study period. This initial analysis compared transplant volume, the rate of NMP utilization, and the proportion of DCD grafts between the “pre‐implementation period” (July 2022–June 2023) and the “post‐implementation period” (July 2023–June 2024).

#### 2.2.2 Part 2: Primary Comparative Analysis in High‐MELD Recipients

For the primary comparative analysis between NMP and SCS, we focused on a cohort of high‐acuity recipients. After excluding 16 DCD and 19 multi‐organ transplants from the total population, we identified patients with a laboratory MELD score of 25 or higher. This resulted in a final study population of 215 isolated DBD liver transplants, which were divided into NMP (*n* = 78) and SCS (*n* = 137) groups based on the preservation method.

#### 2.2.3 Part 3: Subgroup Analysis by CIT

To assess the impact of CIT on outcomes across the entire spectrum of acuity, a subgroup analysis was performed on the total cohort of 299 isolated DBD liver transplants.

Data were collected from our institutional electronic medical record and the Organ Procurement and Transplantation Network (OPTN) database, which is managed by the United Network for Organ Sharing (UNOS).

### 2.3 Outcome Measures and Definitions

The NMP and SCS groups were compared on operative parameters and postoperative clinical outcomes. Operative parameters evaluated included operating room (OR) start time, total operation time, reperfusion‐to‐closure time, intraoperative transfusion volumes, estimated blood loss (EBL), incidence of PRS, and use of open abdominal management. Postoperative clinical outcomes analyzed included laboratory parameters, incidence of EAD, AKI within 48 h after transplantation, postoperative bleeding requiring intervention, mechanical ventilation time, vasopressor requirement time, biliary complications (anastomotic and non‐anastomotic strictures), comprehensive complication index (CCI) at 30 days, and ICU and overall hospital stay.

Liver Donor Risk Index (LDRI) was calculated as defined by Feng et al. [12]. PRS was defined as a decrease in the mean arterial pressure by 30% or more within 5 min following reperfusion and lasting 1 min or longer [13]. EAD was defined according to the criteria proposed by Olthoff et al. [14]. AKI was defined and staged according to the Kidney Disease: Improving Global Outcomes (KDIGO) clinical practice guidelines [15]. Patients who received preoperative or intraoperative dialysis were excluded from the AKI analysis. CCI was calculated according to a previously described and validated methodology [16].

### 2.4 Statistical Analysis

Continuous variables were summarized as medians with interquartile ranges (IQRs) without assuming any specific distributional form. Categorical variables were presented as numbers and percentages. Baseline characteristics of donors and recipients were compared between the NMP and SCS groups using standardized mean differences (SMDs), which quantify between‐group imbalance independently of sample size and are therefore more informative than *p*‐values for descriptive comparisons. Multivariable regression analyses were employed to estimate the associations of NMP versus SCS with operative parameters and postoperative outcomes, adjusting for donor age, CIT, recipient age, and laboratory MELD score. Continuous and binary outcomes were modeled using linear and logistic regression, respectively. Graft survival was analyzed using the Kaplan–Meier method, and survival curves were compared between groups using the Cox proportional hazards model. The confidence level was set to 95%. All statistical analyses were conducted using R ver 4.5.0 (R Core Team, Vienna, Austria).

## 3 Results

### 3.1 Part 1: Institutional Trends Before and After Broad Expansion of NMP Indications

A total of 334 deceased donor liver transplants were performed during the two‐year study period. Comparing the one‐year period before and after the broad expansion of NMP indications, the total number of liver transplants increased from 151 to 183. During these respective periods, NMP utilization rose from 7 cases (4.6%) to 134 (73.2%), and the number of DCD grafts increased from 3 (2.0%) to 13 (7.1%).

### 3.2 Part 2: Baseline Characteristics for the High‐MELD Cohort

**FIGURE 1:**
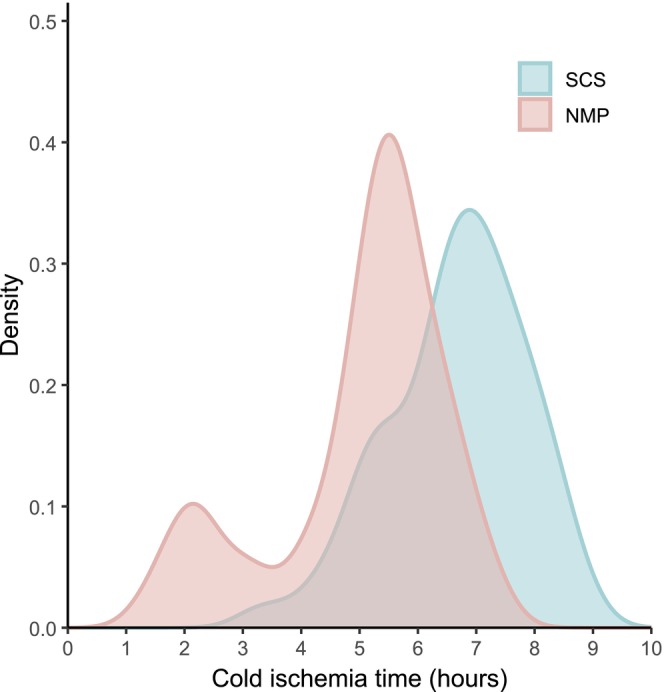
Distribution of cold ischemia time by preservation strategy in the high‐MELD cohort. Overlaid kernel density plots of cold ischemia time (hours) among recipients with MELD ≥ 25, stratified by preservation strategy (SCS, *n* = 137; NMP, *n* = 78). The shaded areas represent kernel density estimates (area normalized to 1) plotted on a common x‐axis scale (0–10 h) to facilitate comparison of distribution shapes between groups.

For the primary analysis, a total of 215 isolated DBD liver transplants in recipients with a MELD score ≥ 25 were analyzed (SCS, *n* = 137; NMP, *n* = 78). Baseline characteristics of donors and recipients for this cohort are summarized in Table 1. Donor characteristics, including age, BMI, laboratory tests, and LDRI, were well‐balanced between the groups. The NMP group had a shorter median CIT compared with the SCS group (5.4 [IQR, 4.7–6.0] vs. 6.8 [IQR, 5.9–7.5] hours). The distribution of CIT in each group is shown in Figure 1 as overlaid kernel density plots. CIT in the SCS group demonstrated a unimodal distribution centered around 6–8 h, whereas the NMP group demonstrated a bimodal distribution with peaks at approximately 2–3 and 5–6 h, consistent with the inclusion of both upfront and back‐to‐base approaches. Recipient characteristics were also largely similar, although recipients in the NMP group tended to be older (median, 55.5 vs. 48 years) and had a higher proportion of patients with hepatocellular carcinoma (11.5% vs. 4.4%).

**TABLE 1:** Baseline characteristics of donors and recipients.

Variables	SCS (*n* = 137)	NMP (*n* = 78)	SMD
Donor characteristics			
Age, y	38 (28–48)	40 (32–48)	0.110
BMI	27.7 (23.8–33.3)	27.8 (24.0–31.8)	0.038
Cause of death, *n* (%)			
Cerebrovascular accident	28 (20.4)	19 (24.4)	0.094
Anoxia	74 (54.0)	44 (56.4)	0.048
Trauma	34 (24.8)	15 (19.2)	0.135
Other	1 (0.7)	0 (0)	0.121
Terminal AST, IU/L	34 (21–70)	37 (21–63)	0.093
Terminal ALT, IU/L	37 (24–92)	40 (19–102)	0.008
Terminal Tbil, mg/dL	0.5 (0.4–0.9)	0.5 (0.3–0.7)	0.172
Macrosteatosis ≥ 30%, *n* (%)	2 (1.5)	1 (1.3)	0.015
LDRI	1.48 (1.32–1.71)	1.52 (1.32–1.69)	0.016
**Preservation parameters**			
NMP Device (Metra/OCS), *n* (%)	NA	63 (80.8)/15 (19.2)	
Back‐to‐Base approach, *n* (%)	NA	62 (79.5)	
Cold ischemia time, h	6.8 (5.9–7.5)	5.4 (4.7–6.0)	
NMP duration, h	NA	10.7 (7.6–12.8)	
Total preservation time, h	6.8 (5.9–7.5)	14.7 (13.0–17.6)	
**Recipient characteristics**			
Age, y	48 (38–61)	55.5 (48–62)	0.367
Sex (Female/Male), *n* (%)	46 (33.6) /91 (66.4)	33 (42.3) /45 (57.7)	0.181
BMI	28.0 (24.0–31.5)	29.3 (25.4–33.0)	0.289
Lab MELD at transplant	35 (30–39)	36 (31–52)	0.181
Pre‐transplant status, *n* (%)			
Not hospitalized	42 (30.7)	29 (37.2)	0.138
Hospitalized, not in ICU	58 (42.3)	25 (32.1)	0.214
ICU	37 (27.0)	24 (30.8)	0.083
Primary diagnosis, *n* (%)			
ALD	97 (70.8)	45 (57.7)	0.276
MASH	19 (13.9)	15 (19.2)	0.145
HCV/HBV	2 (1.5)	5 (6.4)	0.257
PSC/PBC	3 (2.2)	6 (7.7)	0.256
Others	16 (11.7)	7 (9.0)	0.089
HCC, *n* (%)	6 (4.4)	9 (11.5)	0.267
Retransplant, *n* (%)	1 (0.7)	2 (2.6)	0.144

*Note:* Data are presented as median (interquartile range) for continuous variables and numbers (%) for categorical variables. HCC may be present with any primary diagnosis.

Abbreviations: ALD, Alcohol‐related liver disease; ALT, alanine aminotransferase; AST, aspartate aminotransferase; BMI, body mass index; HBV, hepatitis B virus; HCC, hepatocellular carcinoma; HCV, hepatitis C virus; ICU, intensive care unit; LDRI, liver donor risk index; MASH, metabolic dysfunction‐associated steatotic hepatitis; MELD, model for end‐stage liver disease; NA, not applicable; NMP, normothermic machine perfusion; PBC, primary biliary cholangitis; PSC, primary sclerosing cholangitis; SCS, static cold storage; SMD, standardized mean difference; Tbil, total bilirubin.

#### 3.2.1 Operative Parameters

Operative parameters are summarized in Table 2. NMP was associated with a significantly higher likelihood of a daytime operating room start (8:00 a.m.–5:00 p.m.) compared with SCS (88.5% vs. 23.4%; adjusted OR, 45.7; 95% CI, 17.0–143.3). The NMP group had a modestly longer total operation time than the SCS group (median, 345 vs. 321 min; adjusted mean difference, 34.1 min; 95% CI, 6.9–61.3). However, the interval from reperfusion to the end of surgery was similar between the groups. The incidence of PRS, EBL, and intraoperative blood product transfusion volumes were comparable.

**TABLE 2:** Operative parameters.

Variables	SCS (*n* = 137)	NMP (*n* = 78)	OR* (95% CI)
Post‐reperfusion syndrome, *n* (%)	54 (39.4)	23 (29.5)	0.72 (0.35–1.46)
Open abdominal management, *n* (%)	9 (6.6)	7 (9.0)	0.91 (0.25–3.16)
Daytime OR start^†^, *n* (%)	32 (23.4)	69 (88.5)	45.7 (17.0–143.3)
Variables	SCS (*n* = 137)	NMP (*n* = 78)	Mean difference* (95% CI)
Total operation time, min	321 (263–370)	345 (293–400)	34.1 (6.9–61.3)
Reperfusion to end of surgery time, min	189 (153–227)	197 (155–240)	8.7 (−11.9–29.3)
Blood product transfusion			
RBC, mL	1550 (930–2480)	1828 (1240–2713)	278.5 (−234.3–791.3)
FFP, mL	2001 (1117–3008)	2524 (1571–3609)	536.0 (−95.4–1167.3)
Cryoprecipitate, mL	205 (94–356)	226 (93–367)	−22.8 (−91.1–45.6)
Platelet, mL	338 (0–719)	526 (0–872)	−9.2 (−184.2–165.7)
EBL, mL	2500 (1300–4700)	3850 (1763–5763)	1163.9 (−722.0–3049.7)

*Note:* Data are presented as median (interquartile range) for continuous variables and numbers (%) for categorical variables.

Abbreviations: CI, confidence interval; EBL, estimated blood loss; FFP, fresh frozen plasma; NMP, normothermic machine perfusion; OR, odds ratio/operating room; RBC, red blood cells; SCS, static cold storage.

* Odds ratios and mean differences were adjusted for donor age, cold ischemia time, recipient age, and laboratory MELD score.

^†^ Daytime operating room start was defined as a surgical start time between 8:00 a.m. and 5:00 p.m.

#### 3.2.2 Postoperative Outcomes

Postoperative outcomes are summarized in Table 3. The incidences of major clinical outcomes were similar between the groups. Specifically, there were no significant differences in postoperative bleeding, EAD (17.9% vs. 24.1%; adjusted OR, 0.86; 95% CI, 0.36–1.97), AKI, or biliary complications.

**TABLE 3:** Postoperative outcomes.

Variables	SCS (*n* = 137)	NMP (*n* = 78)	OR* (95% CI)
Postoperative bleeding	19 (13.9)	7 (9.0)	0.52 (0.16–1.49)
EAD	33 (24.1)	14 (17.9)	0.86 (0.36–1.97)
AKI within 48 h^†^	25/59 (42.4)	18/39 (46.2)	0.93 (0.33–2.55)
Anastomotic biliary stricture	23 (16.8)	19 (24.4)	1.44 (0.63–3.27)
Non‐anastomotic biliary stricture	1 (0.7)	2 (2.6)	3.11 (0.19–84.31)
Variables	SCS (*n* = 137)	NMP (*n* = 78)	Mean difference* (95% CI)
Peak AST, IU/L	1112 (635–2024)	623 (331–1251)	−367.5 (−913.8–178.7)
Peak ALT, IU/L	653 (355–1150)	298 (154–625)	−298 (−566.5– −29.6)
Tbil on POD 7, mg/dL	3.3 (2.0–6.8)	3.2 (1.5–5.7)	−0.8 (−2.2–0.6)
Ventilator time, h	17.5 (8.0–34.1)	21.6 (15.5–47.6)	80.9 (−26.3–188.0)
Pressor requirement time, h	0.0 (0.0–12.4)	6.5 (0.0–23.1)	5.9 (−6.5–18.3)
CCI at 30 days	29.6 (20.9–42.7)	26.2 (0.0–44.8)	−7.5 (−15.3–0.3)
ICU stay, d	4.0 (3.0–6.0)	6.5 (4.0–14.2)	5.2 (−0.5–10.8)
Hospital stay, d	16.0 (10.0–27.0)	19.0 (13.0–42.5)	1.5 (−8.4–11.5)

*Note:* Data are presented as median (interquartile range) for continuous variables and numbers (%) for categorical variables.

Abbreviations: AKI, acute kidney injury; ALT, alanine aminotransferase; AST, aspartate aminotransferase; CCI, comprehensive complication index; CI, confidence interval; EAD, early allograft dysfunction; ICU, intensive care unit; MELD, model for end‐stage liver disease; NMP, normothermic machine perfusion; OR, odds ratio; POD, postoperative day; SCS, static cold storage; Tbil, total bilirubin.

* Odds ratios and mean differences were adjusted for donor age, cold ischemia time, recipient age, and laboratory MELD score.

^†^ Patients who received preoperative or intraoperative dialysis were excluded from the AKI analysis.

**FIGURE 2:**
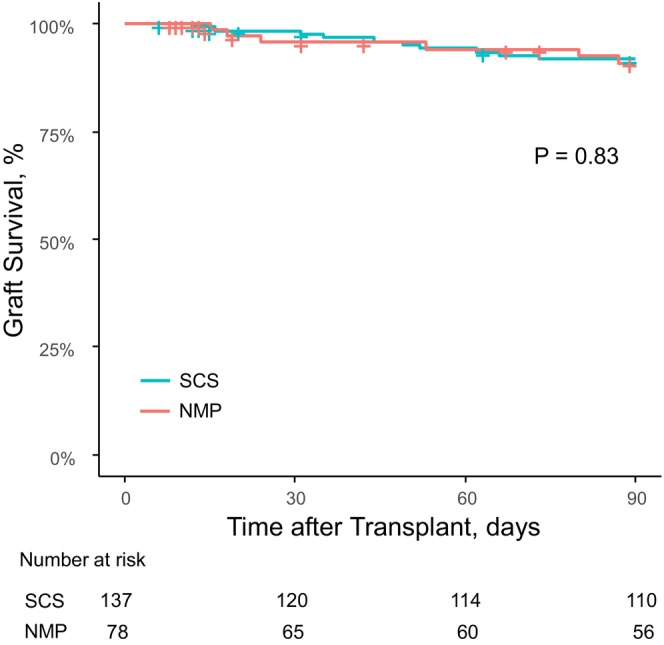
Ninety‐day graft survival in the high‐MELD cohort. Kaplan–Meier analysis of graft survival during the first 90 days post‐transplantation in recipients with a laboratory MELD score ≥ 25. There was no significant difference in survival between the normothermic machine perfusion (NMP) and static cold storage (SCS) groups (*p* = 0.83, log‐rank test).

In the analysis of continuous outcomes, peak alanine aminotransferase (ALT) was significantly lower in the NMP group (median 298 vs. 653 IU/L; adjusted mean difference −298 IU/L; 95% CI, −566.5 to −29.6). However, other parameters such as peak aspartate aminotransferase (AST), total bilirubin on postoperative day 7, ventilator time, pressor requirement time, ICU stay, and total hospital stay were similar between the groups. Ninety‐day graft survival was 91.8% in the SCS group and 90.9% in the NMP group, with no significant difference between groups (Hazard ratio, 1.11; 95% CI, 0.41–3.07; Figure 2).

### 3.3 Part 3: Subgroup Analysis by CIT

**FIGURE 3:**
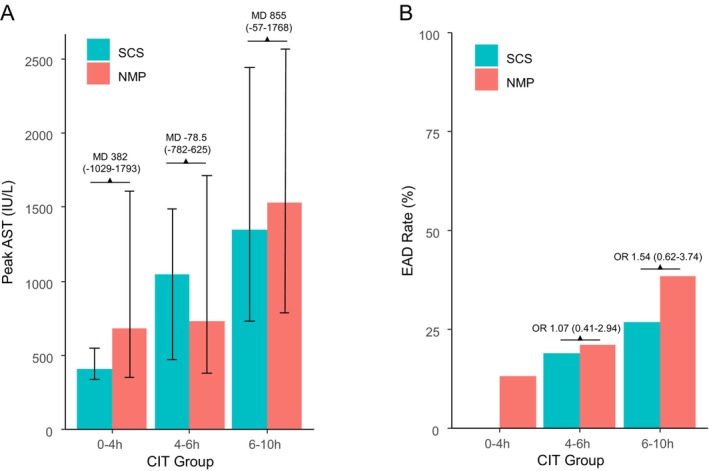
Impact of cold ischemia time on postoperative outcomes. (A) Mean peak AST levels and (B) incidence of EAD stratified by CIT categories for the NMP and SCS groups. While no significant differences were observed between NMP and SCS within the same CIT category, a pooled analysis showed that longer CIT was associated with significantly higher peak AST levels and a greater incidence of EAD. Error bars represent 95% confidence intervals. AST, aspartate aminotransferase; EAD, early allograft dysfunction; CIT, cold ischemia time; MD, mean difference; NMP, normothermic machine perfusion; OR, odds ratio; SCS, static cold storage.

To investigate the impact of CIT on outcomes, a subgroup analysis was performed on the entire cohort of 299 isolated DBD transplants, comparing peak AST levels and EAD incidence between NMP and SCS within three CIT categories (0–4, 4–6, and 6–10 h). No significant differences in peak AST levels or EAD incidence were observed between NMP and SCS within the same CIT category (Figure 3A,B). However, in a pooled analysis combining both preservation methods, the duration of CIT itself emerged as a significant predictor of worse outcomes. Specifically, a linear regression demonstrated that increasing CIT was associated with higher peak AST (slope, 251 IU/L per hour; 95% CI, 105.0–397.2). Similarly, longer CIT was associated with a higher incidence of EAD, as shown by the Cochran–Armitage trend test (*p* = 0.03).

## 4 Discussion

**FIGURE 4:**
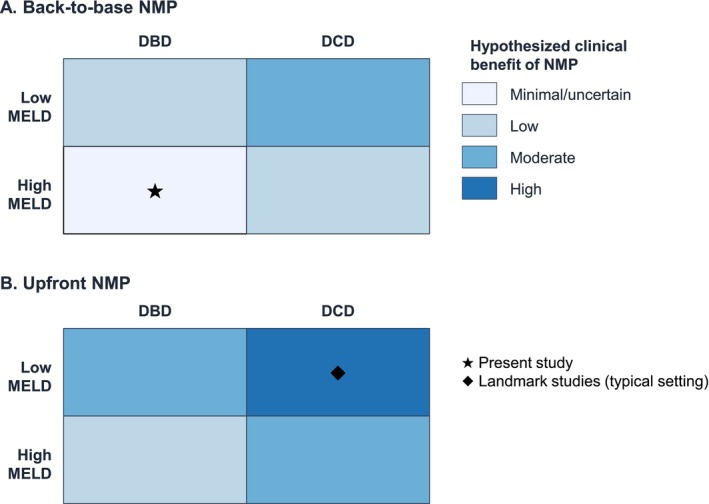
Conceptual framework of the hypothesized clinical benefit of NMP by graft type, perfusion strategy, and recipient acuity. Heatmaps show back‐to‐base NMP (A) and upfront NMP (B), stratified by graft type (DBD vs DCD) and recipient acuity (low MELD vs high MELD). Darker shading indicates greater hypothesized clinical benefit, and lighter shading indicates minimal/uncertain benefit. The star denotes the setting of the present study (DBD, high‐acuity recipient, back‐to‐base NMP). The diamond denotes the typical setting of landmark NMP studies (DCD, low‐acuity recipient, upfront NMP). This is a conceptual figure and does not represent quantitative estimates. DBD, donation after brain death; DCD, donation after circulatory death; MELD, Model for End‐Stage Liver Disease; NMP, normothermic machine perfusion.

We evaluated the utility of NMP, primarily utilizing a back‐to‐base approach, in a unique cohort of high‐MELD recipients receiving DBD grafts. Accordingly, our findings may be particularly informative for programs in Japan and other parts of East Asia, where DCD utilization and routine upfront portable perfusion are not yet widely available. The finding of a lower peak ALT in the NMP group should be interpreted with caution. Because perfusate AST/ALT levels were not routinely measured in our cohort, it remains unclear whether this difference is primarily attributable to a “washout effect” into the perfusion circuit or reflects a true reduction in hepatocellular injury. Future studies incorporating perfusate transaminase measurements may help clarify the contribution of enzyme clearance during NMP [17]. Aside from this single biochemical parameter, our analysis found no significant differences in other major short‐term clinical outcomes, such as EAD, AKI, CCI, hospital stay, and 90‐day graft survival. These findings contrast with much of the existing literature and underscore that the clinical benefit of NMP is context‐dependent. Accordingly, interpretation of NMP studies should account for three interacting determinants: graft type (DBD vs. DCD), preservation strategy (back‐to‐base vs. upfront), and recipient acuity. To aid readers, a summary of major NMP studies is provided in Table 4, and a conceptual framework summarizing these interactions and highlighting the typical setting of landmark NMP studies (DCD, low‐acuity recipients, upfront NMP) as well as the setting of the present study (DBD, high‐acuity recipients, back‐to‐base NMP) is shown in Figure 4. The following discussion is structured around these three perspectives.

**TABLE 4:** Summary of major NMP studies highlighting preservation logistics, graft type, and recipient acuity.

Study	Design/NMP logistics & device	Donor type and number of cases	Recipient MELD	Key short‐term outcomes (stratified by donor type when available)
Nasralla et al. (2018, Nature)	Multicenter RCT/Upfront NMP (Metra)	NMP: 121 (DBD 87, DCD 34), SCS: 101 (DBD 80, DCD 21)	Not stratified by donor type. NMP 13 (10–18) vs. SCS 14 (9–18)	DBD: Reduced peak AST by 40.2%. DCD: Reduced peak AST by 73.3%. Overall: Reduced EAD (72% lower odds).
Markmann et al. (2022, JAMA Surg)	Multicenter RCT/Upfront NMP (OCS)	NMP: 151 (DBD 123, DCD 28), SCS: 142 (DBD 131, DCD 11)	Not stratified by donor type. NMP 28.4 ± 6.9 vs. SCS 28.0 ± 5.7	Overall: Reduced EAD (18% vs. 31%).
Chapman et al. (2023, Ann Surg)	Multicenter RCT/Upfront NMP (Metra)	NMP: 136 (DBD 114, DCD 22), SCS: 130 (DBD 114, DCD 16)	DBD‐NMP 19.6 ± 9.9 vs. DBD‐SCS 19.3 ± 9.3 DCD‐NMP 17.3 ± 7.0 vs. DCD‐SCS 20.3 ± 4.7	Overall: Reduced peak AST (540.3 vs. 722.4 IU/L); no difference in EAD.
Yamamoto et al. (2023, Ann Surg)	Single‐center retrospective cohort/Upfront NMP (OCS)	NMP: 110 (DBD 58, DCD 52), SCS: 431 (DBD 411, DCD 20)	DBD‐NMP 25.1 ± 12.5 vs. DBD‐SCS 26.8 ± 12.1. DCD‐NMP 19.3 ± 8.2 vs. DCD‐SCS 18.2 ± 5.9	DBD: No difference in EAD or PRS. DCD: Reduced EAD (25.0% vs. 65.0%); Reduced PRS (16.0% vs. 78.9%).
Wehrle et al. (2024, Ann Surg)	Multicenter retrospective cohort/Back‐to‐base NMP (Metra)	NMP: 155 (DBD 118, DCD 37). SCS: 310 (DBD 236, DCD 74)	DBD‐NMP 19 (14–27) vs. DBD‐SCS 21 (13–29.3). DCD‐NMP 19 (13–21) vs. DCD‐SCS 20 (12–29)	DBD: No difference in CCI. DCD: Reduced 90‐day CCI (27.6% vs. 41.9%). Overall: Reduced need for early relaparotomy and RRT. EAD was not reported.
Gao et al. (2025, Liver Transpl)	Single‐center retrospective cohort/Upfront NMP (OCS)	NMP: 144 (DBD 89, DCD 55). SCS: 149 (DBD 142, DCD 7)	Not stratified by donor type. NMP 22 (17–26) vs. SCS 26 (18–32)	Overall: Reduced PRS (4.3% vs. 32.9%); less blood loss (1.5 vs. 3.0 L); shorter ICU stay (3 vs. 5 days) and hospital stay (11 vs. 13 days). No difference in EAD.
Nguyen et al. (2025, JAMA Surg)	Single‐center retrospective cohort/Upfront NMP (OCS)	NMP: 342 (DBD 63, DCD 279), SCS: 744 (DBD 480, DCD 264)	DBD‐NMP 19 (11–26) vs. DBD‐SCS 22 (16–29). DCD‐NMP 19 (13–23) vs. DCD‐SCS 21 (17–23)	DBD: No difference in EAD; similar graft survival. DCD: Reduced EAD (17.5% vs. 50.0%); lower graft failure risk (HR 0.13).
Present study (Sambommatsu et al.)	Single‐center retrospective cohort/Predominantly back‐to‐base NMP (Metra/OCS)	NMP: 78 (DBD 78, DCD 0), SCS: 137 (DBD 137, DCD 0)	DBD‐NMP 36 (31–52) vs. DBD‐SCS 35 (30–39)	DBD: Reduced peak ALT (298 vs. 653 IU/L); no difference in EAD or PRS.

*Note:* MELD is reported as presented in each study (mean ± SD or median [IQR]).

Abbreviations: ALT, alanine aminotransferase; AST, aspartate aminotransferase; CCI, Comprehensive Complication Index; DBD, donation after brain death; DCD, donation after circulatory death; EAD, early allograft dysfunction; HR, hazard ratio; ICU, intensive care unit; IQR, interquartile range; MELD, Model for End‐Stage Liver Disease; NMP, normothermic machine perfusion; OCS, Organ Care System; PRS, post‐reperfusion syndrome; RCT, randomized controlled trial; RRT, renal replacement therapy; SCS, static cold storage.

Many studies reporting the superiority of NMP have included a high proportion of DCD grafts, and subgroup analyses consistently demonstrate that the clinical benefits are most pronounced in DCD grafts, while effects in DBD grafts are often limited (Table 4). For example, a large multicenter study by Nguyen et al. found that NMP significantly reduced the incidence of EAD in DCD grafts (17.5% vs. 50.0%; *p* = 0.002), but not in DBD grafts (36.8% vs. 27.3%; *p* = 0.56) [6]. Similarly, a large single‐center report by Yamamoto et al. identified NMP as an independent protective factor against EAD in DCD transplants (OR 0.11; 95% CI, 0.03–0.46; *p* < 0.01), but not in their DBD cohort.

Consistent with these reports, our finding of no significant difference in short‐term outcomes between NMP and SCS in DBD grafts suggests that the protective effect of NMP in this setting may be limited. This discrepancy likely stems from the fundamental difference in ischemic injury between graft types. DCD grafts are inevitably exposed to a significant period of warm ischemic injury during the agonal phase, a primary driver of subsequent severe ischemia–reperfusion injury [18]. NMP is particularly effective at mitigating this cellular damage by restoring metabolic function and replenishing ATP levels during perfusion [1]. In contrast, standard DBD grafts do not experience this severe warm ischemic insult. Consequently, the baseline level of injury in standard DBD grafts is inherently lower, creating a potential “ceiling effect” where the margin for NMP to demonstrate a significant improvement over conventional SCS is limited.

Another crucial aspect is the NMP application model. Most landmark studies utilized an upfront approach, which minimizes CIT by initiating perfusion at the donor hospital (Table 4), [1, 2, 4, 6]. In contrast, our study predominantly reflects a back‐to‐base approach (79.5% of NMP cases), which, while logistically simpler, involves an initial period of SCS and resulted in a median CIT that was only 1.4 h shorter than in the SCS group. To date, the study by Wehrle et al. is the only other large‐scale investigation (with > 100 NMP cases) comparing back‐to‐base NMP with SCS [8]. While they did not report on EAD, their primary outcome, CCI, showed no superiority for NMP in DBD grafts, though a benefit was observed in DCD grafts. Our study aligns with their findings, as we similarly did not demonstrate the superiority of NMP for major short‐term outcomes in DBD grafts.

Our subgroup analysis further clarifies the impact of CIT. While no significant differences in EAD or peak AST levels were found between NMP and SCS within the same CIT categories, a pooled analysis of both preservation methods demonstrated that longer CIT was associated with worse outcomes. This finding confirms the well‐established detrimental impact of prolonged CIT in SCS‐preserved livers and extends this observation to grafts preserved with NMP [19]. Supporting this, a recent study also demonstrated that a prolonged CIT before the start of NMP is an independent risk factor for EAD in DCD transplants [9]. Our study is the first to show a similar association in DBD grafts. These results suggest longer CIT is associated with an increased EAD risk regardless of the preservation method, and the initial cold ischemic damage may not be entirely reversible by subsequent NMP.

Finally, recipient acuity is a critical differentiating factor. Whereas the existing literature has predominantly focused on recipients with median or mean MELD ranging from 15 to 28, where graft quality may be a primary driver of outcomes, our study is the first to report on a cohort with a median MELD score of 35 or higher (Table 4), [1, 2, 4, 5, 6, 7, 8, 20, 21]. It is well recognized that transplantation in high‐MELD patients is associated with greater technical challenges and a higher risk of poor outcomes due to factors such as severe portal hypertension, profound coagulopathy, and concomitant multi‐organ dysfunction. In these severely ill patients, it is plausible that postoperative outcomes are driven more strongly by these recipient‐related factors than by the incremental benefits of NMP, potentially masking any advantages that are more readily apparent in lower‐acuity populations.

Nonetheless, NMP confers other benefits beyond the primary endpoints of this study. As our data demonstrated, a major advantage lies in logistical optimization, enabling the transition of transplants to daytime hours [3, 22]. This shift may reduce surgeon and operating room staff fatigue and improve well‐being, a topic our group is currently investigating. Furthermore, for cases requiring prolonged recipient hepatectomies, such as re‐transplantations or those with extensive adhesions, NMP alleviates the pressure of CIT constraints. Finally, the ability to perform viability assessment during perfusion facilitates a more aggressive organ acceptance strategy. This, in turn, can lead to an increased utilization of DCD grafts and a growth in overall transplant volume, a trend also observed at our institution [23].

This study has limitations. First, as a single‐center, retrospective, non‐randomized study, our analysis remains vulnerable to unmeasured confounding; however, after July 2023 NMP became the default preservation strategy at our center, reducing the likelihood of case‐by‐case preferential assignment and thereby lessening concerns regarding selection bias. Second, given the sample size and event rates, our study may have been underpowered to detect modest but clinically meaningful differences between preservation strategies; larger cohorts may better delineate such effects. Third, the findings may not be generalizable to centers with different donor‐recipient characteristics or NMP operational protocols. Fourth, higher‐risk donor grafts (e.g., higher age or grafts with steatosis) were uncommon in our cohort, precluding meaningful subgroup analyses. Finally, this study was limited to short‐term outcomes, and future longer‐term follow‐up is required.

In conclusion, in the specific setting of DBD grafts transplanted into high‐MELD recipients, NMP performed predominantly with a back‐to‐base approach did not improve short‐term outcomes compared with SCS. The clinical benefits of NMP appear to be context‐dependent, with the greatest advantages likely realized in higher‐risk grafts using an upfront perfusion strategy. Interpreting NMP studies requires careful consideration of three critical factors: graft type, application model (upfront vs. back‐to‐base), and recipient acuity.

## Funding

The authors have nothing to report.

## Ethics Statement

This study was approved by the Institutional Review Board of Virginia Commonwealth University (HM20029955).

## Consent

The authors have nothing to report.

## Conflicts of Interest

I.Y. reports grants from KAKENHI and AMED and speaker fees from Chugai Pharmaceutical Co. and Pfizer outside the submitted work. The other authors declare no conflicts of interest.

## Supporting information


**Table S1:** Institutional criteria for considering static cold storage (SCS) as an exception to default back‐to‐base normothermic machine perfusion (NMP) since July 2023.

## Data Availability

The data that supports the findings of this study are available in the Supporting Information of this article.
