# Supplementary material for: Normothermic Machine Perfusion vs. Static Cold Storage for DBD Liver Transplantation in High‐MELD Recipients: A Single‐Center Retrospective Study
Source: J Hepatobiliary Pancreat Sci. 2026 May 8;33(8):605–14. doi: 10.1002/jhbp.70129 (PMC13485208; doi:10.1002/jhbp.70129)
Supplement: Supplementary file 1 [file JHBP-33-605-s001.docx]

SUPPLEMENTAL TABLE 1. Institutional criteria for considering static cold storage (SCS) as an exception to default back-to-base normothermic machine perfusion (NMP) since July 2023

| **Domain** | **Criteria for considering SCS (all must be met)** |
| --- | --- |
| Donor (standard-criteria graft) | Age ≤60; macrosteatosis ≤20%; no significant hemodynamic instability; no prolonged vasopressor requirement; acceptable allocation laboratory profile |
| Recipient (clinically straightforward) | Recipient age <70; MELD <30; No redo LT; no PVT; BMI <40; no significant cardiac disease |
| Logistics (timing/arrival) | Expected early-morning graft arrival enabling daytime transplantation without schedule extension |

Back-to-base NMP using OrganOx Metra was the default preservation strategy at our center from July 2023 onward; SCS was considered only when all criteria across donor, recipient, and logistics domains were satisfied. When a local recovery surgeon was unavailable, TransMedics Organ Care System (OCS) was used as an alternative preservation strategy.

BMI, body mass index; LT, liver transplantation; MELD, Model for End-stage Liver Disease; NMP, normothermic machine perfusion; PVT, portal vein thrombosis; SCS, static cold storage.
